# Suspected cumulative neuro-ophthalmic toxicity after chronic oral exposure to the veterinary anesthetic tiletamine: a case report

**DOI:** 10.3389/fvets.2026.1789169

**Published:** 2026-04-08

**Authors:** Ligang Jiang, Xin Jiang, Ruixuan Xu, Weihua Yang, Yuhua Tong

**Affiliations:** 1 Department of Ophthalmology, Quzhou Affiliated Hospital of Wenzhou Medical University, Quzhou People's Hospital, Quzhou, Zhejiang, China; 2 Quzhou College of Technology, Quzhou, Zhejiang, China; 3 Clinical Medical College, Hainan Medical University, Haikou, China; 4 Shenzhen Eye Hospital, Shenzhen Eye Medical Center, Southern Medical University, Shenzhen, Guangdong, China

**Keywords:** case report, neurotoxicity, One Health, Telazol, tiletamine, veterinary anesthetics, visual dysfunction, Zoletil

## Abstract

**Background:**

Tiletamine, commonly formulated with zolazepam as Zoletil^®^/Telazol^®^, is widely used in veterinary anesthesia; however, human data regarding potential cumulative neuro-ophthalmic toxicity after long-term exposure remain limited.

**Case presentation:**

A 24-year-old man developed progressive bilateral visual decline (left worse than right) after >1 year of non-medical oral exposure to a suspected tiletamine-containing product obtained online. He also reported persistent dyschromatopsia, dysarthria, and gait instability after cessation. Best-corrected visual acuity was 20/25 (right) and 20/50 (left). Fundus and ultra-widefield imaging were unremarkable. Optical coherence tomography showed bilateral fovea-centered outer retinal/photoreceptor-layer disruption with scattered hyperreflective foci. Full-field electroretinography was within normal limits, whereas visual evoked potentials revealed reduced P100 amplitude in the left eye with preserved latency. Brain MRI demonstrated mild diffuse cerebral and cerebellar sulcal widening compatible with early atrophy. Alternative causes, including inherited retinal degeneration and other established retinotoxic exposures, were not supported by the available evaluation. Short-term follow-up showed clinical stability with limited recovery.

**Conclusion:**

This case suggests a possible temporal association between prolonged oral exposure to a suspected tiletamine-containing product and combined retinal and neurological abnormalities. Because toxicological confirmation and independent formulation verification were unavailable, causality cannot be established. The findings highlight the need for One Health–oriented toxicovigilance and longitudinal neuro-ophthalmic monitoring when similar exposure is suspected.

## 1 Introduction

Tiletamine is an arylcyclohexylamine dissociative anesthetic structurally similar to ketamine. It is primarily used in veterinary anesthesia, most commonly as a fixed combination with the benzodiazepine zolazepam marketed as Zoletil^®^ or Telazol^®^ (1, 2). As a widely used veterinary anesthetic, tiletamine-containing products are encountered in routine clinical practice, wildlife/field immobilization, and laboratory animal procedures, creating potential opportunities for accidental exposure, diversion, or non-medical use at the animal–human interface. In this fixed combination, tiletamine provides dissociative anesthesia and analgesia, whereas zolazepam contributes anxiolysis, muscle relaxation, and attenuation of emergence reactions. Tiletamine has a rapid onset and provides dissociative anesthesia with analgesia; in contrast, the “muscle relaxation” effect commonly attributed to tiletamine-containing products is largely mediated by zolazepam, while tiletamine itself may be associated with increased muscle tone/rigidity in some settings (3). However, owing to safety considerations, tiletamine has not been approved for human use. Reported adverse effects may involve the cardiovascular, respiratory, and central nervous systems, including sympathomimetic excitation, respiratory depression, hypersalivation, delayed emergence, muscle hypertonia/rigidity, central nervous system excitation, seizures, and pulmonary edema; fatal outcomes have also been reported in cases of overdose or misuse (4, 5). From a veterinary pharmacology and toxicology perspective, characterizing such adverse outcomes is essential for toxicovigilance, occupational safety, and risk communication.

Given that tiletamine is an N-methyl-D-aspartate (NMDA) receptor antagonist, acute low-dose exposure may, in certain experimental contexts, transiently reduce excitotoxicity and confer short-lived neuroprotective effects (6–8). In contrast, prolonged or repeated exposure may disrupt NMDA receptor–mediated neurotransmission and synaptic plasticity, thereby perturbing neural network homeostasis and weakening the functional stability of the visual pathway (9). Moreover, NMDA receptor dysfunction itself has been associated with visual symptoms (10). Although there is currently no direct evidence that tiletamine causes retinal functional impairment or structural disruption of photoreceptors, the possibility of chronic exposure–related neuro-ophthalmic toxicity merits attention, particularly given the limited human safety data for veterinary anesthetics. Existing evidence on tiletamine-related adverse effects is derived predominantly from veterinary observations and preclinical studies (11, 12).

Most reports have focused on complications observed in acute veterinary anesthesia, including ataxia, cardiopulmonary instability, abnormal emergence behaviors, and prolonged sedation (13–16). In humans, the available evidence remains limited, with only a small number of case reports describing adverse events associated with tiletamine or tiletamine-containing formulations (5, 17–20), primarily in the context of accidental exposure, misuse, or abuse. These cases have most often involved injection and relatively short exposure durations, and clinical manifestations have largely been acute, such as respiratory depression, seizures, and central nervous system excitation. However, most published cases lack long-term follow-up and systematic neuroimaging data, and visual involvement has rarely been described. Against this background, well-documented case reports are particularly valuable. Reporting rare or unexpected adverse effects not only helps expand the current knowledge base but also provides important clinical warnings for human medicine and occupational safety in veterinary practice. Importantly, such reports may also inform surveillance strategies and regulatory considerations related to access and misuse of veterinary anesthetic agents.

To our knowledge, reports linking the veterinary anesthetic tiletamine to retinopathy accompanied by central nervous system involvement remain exceedingly rare. Here, we describe a patient who developed progressive visual dysfunction after long-term oral tiletamine use, with neuroimaging findings suggestive of brain atrophy. This case expands the clinical spectrum of tiletamine-associated adverse effects and highlights the possibility of under-recognized neurotoxicity with chronic exposure, with important implications for clinical surveillance and drug regulation. By documenting multimodal retinal imaging, electrophysiological abnormalities, and neuroimaging findings in the setting of prolonged exposure, this report provides a clinically relevant safety signal supporting toxicovigilance at the animal–human interface.

## 2 Case presentation

A 24-year-old man was admitted with progressive bilateral blurred vision for 6 months, with worsening over the preceding 2 months. At symptom onset, he noted gradual visual blurring accompanied by mild dysarthria and gait unsteadiness, which he did not seek medical attention for. As the disease progressed, visual acuity declined further. He subjectively reported dim vision with dyschromatopsia (a “greenish” tint), more pronounced in the left eye. He also developed impaired speech expression affecting communication and an abnormal gait consistent with cerebellar ataxia (unsteady gait and reduced coordination of movements). He was previously healthy and denied a history of chronic systemic diseases, including hypertension, diabetes mellitus, or coronary artery disease. He reported no smoking or alcohol use, and denied a family history of neurological disorders or inherited retinal degenerative diseases.

Further history taking revealed that, 1 year earlier, due to academic stress and psychological strain, he had obtained a veterinary anesthetic, tiletamine, via online sources and used it orally for a prolonged period. He took it at irregular intervals, up to six times per day, at an estimated dose of 200–300 mg per intake, for approximately 1 year, and had discontinued it 2 months before admission. He denied concomitant use of other drugs known to cause retinal toxicity. Notably, the exact formulation of the suspected agent could not be independently verified. Although the patient described the product as “tiletamine,” we could not exclude the possibility that it represented a compounded preparation, adulterated material, or a tiletamine–zolazepam combination (e.g., Zoletil/Telazol), given its veterinary availability and potential variability across informal supply chains. At presentation, no remaining sample, packaging, batch information, or purchase records were available for verification. Toxicological confirmation (e.g., LC–MS/MS in blood/urine or chemical analysis of residual material) was not performed because the patient presented after cessation and no specimen was available. Therefore, the exposure details should be interpreted as patient-reported estimates and may be subject to recall bias and potential misclassification of the exact compound(s).

**Figure 1:**
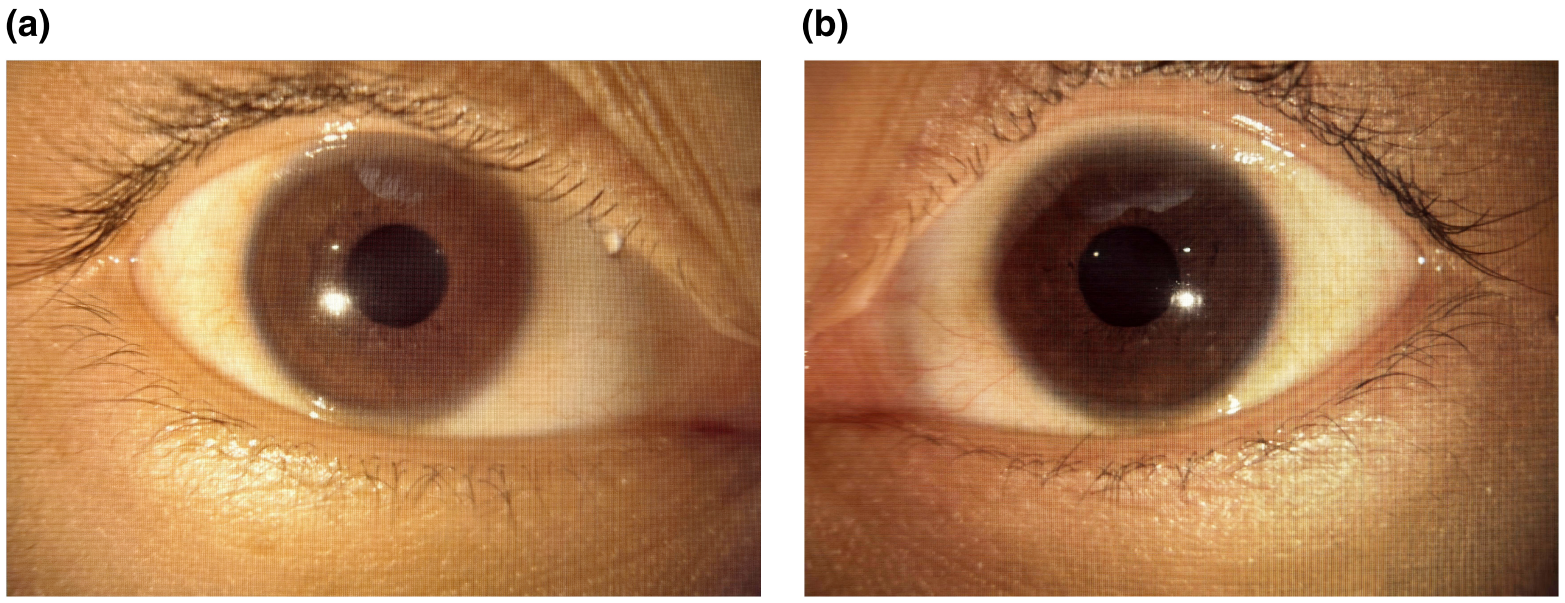
Anterior segment photographs. Anterior segment examination shows a largely quiet anterior segment with mild perilimbal/perikeratic conjunctival injection, clear corneas, deep and quiet anterior chambers, round pupils (~3 mm) with preserved light reflexes, and no obvious lens opacities. **(a)** Right eye. **(b)** Left eye.

**Figure 2:**
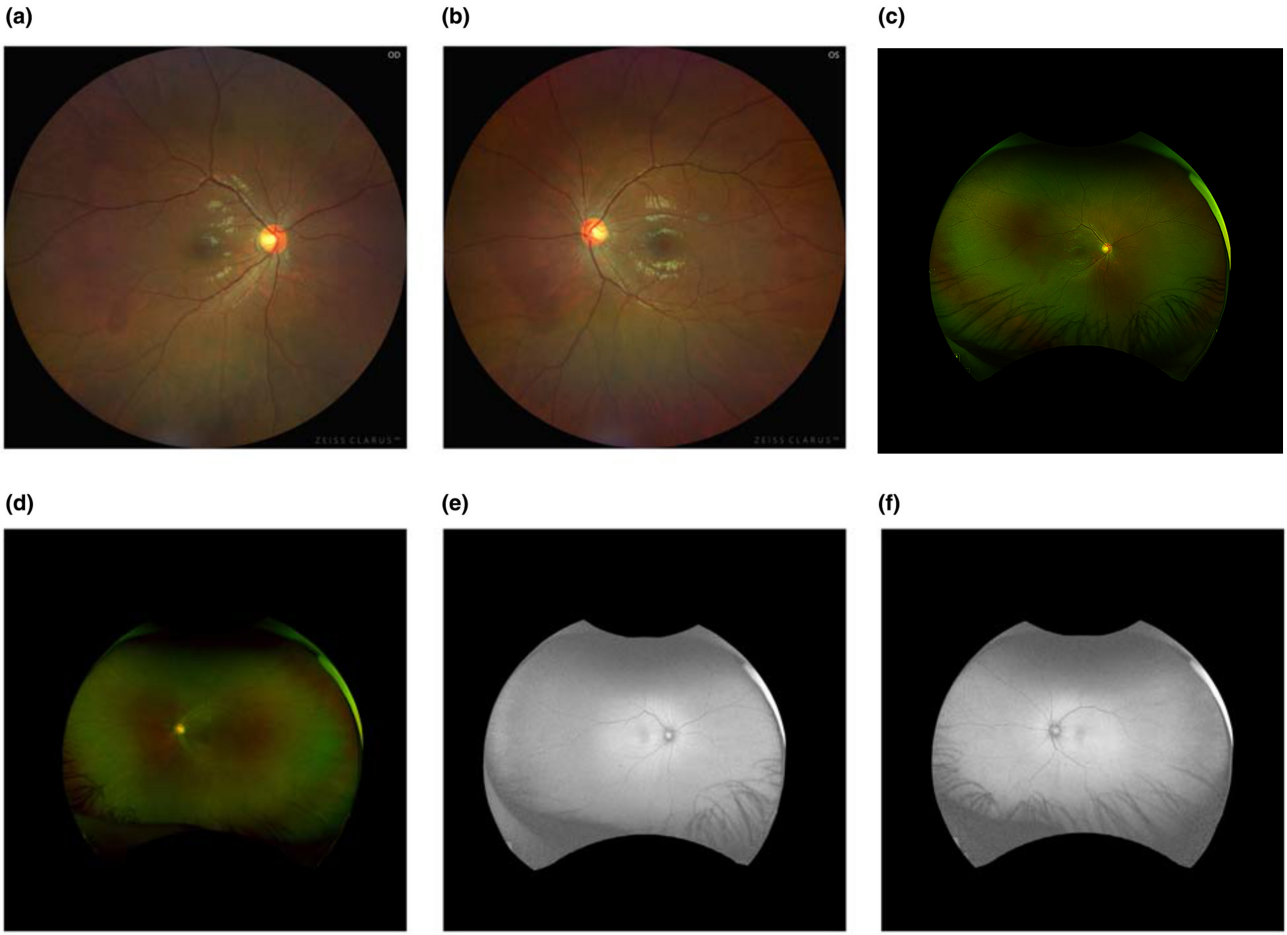
Fundus imaging and fundus autofluorescence. Color fundus photography and ultra-widefield imaging reveal no definite abnormalities in the posterior pole or peripheral retina. Ultra-widefield fundus autofluorescence shows no abnormal hypo- or hyperautofluorescent lesions. **(a)** Color fundus photograph of the right eye. **(b)** Color fundus photograph of the left eye. **(c)** Ultra-widefield fundus image of the right eye. **(d)** Ultra-widefield fundus image of the left eye. **(e)** Ultra-widefield fundus autofluorescence of the right eye. **(f)** Ultra-widefield fundus autofluorescence of the left eye.

**Figure 3:**
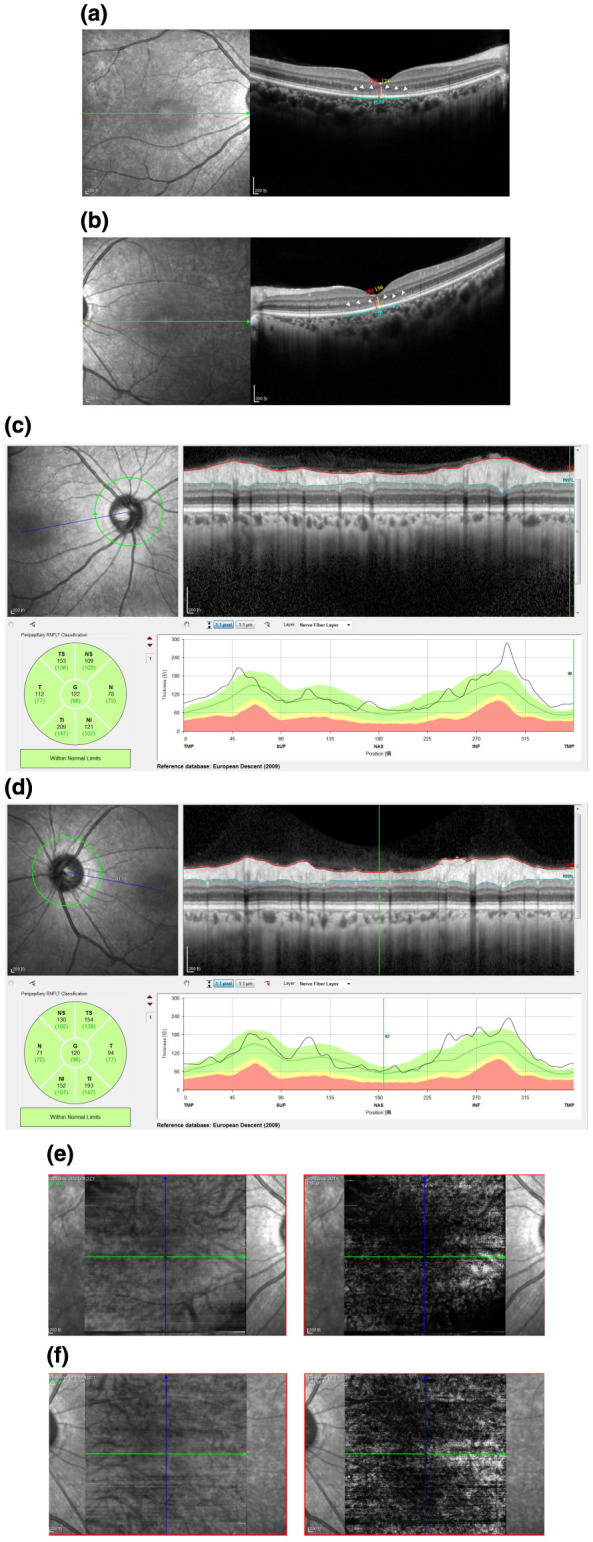
Multimodal retinal imaging findings (OCT, OCTA, and RNFL analysis). Macular OCT demonstrates bilateral, fovea-centered outer retinal/photoreceptor abnormalities with irregularity and partial disruption of photoreceptor-related bands and scattered hyperreflective foci/deposit-like changes (white arrowheads). CMT and ORT were measured on the fovea-centered scans; the red vertical line indicates CMT, the yellow vertical line indicates ORT, and the blue horizontal line indicates the linear extent of the affected outer retinal segment. Quantitative measurements show CMT of 184 μm in the right eye and 180 μm in the left eye, ORT of 124 μm in the right eye and 136 μm in the left eye, and an affected-segment length of approximately 636 μm in the right eye and 720 μm in the left eye. Peripapillary RNFL thickness maps show no definite thinning. OCTA shows no evidence of macular neovascularization **(a)** Macular OCT B-scan of the right eye. **(b)** Macular OCT B-scan of the left eye. **(c)** Peripapillary RNFL analysis of the right eye. **(d)** Peripapillary RNFL analysis of the left eye. **(e)** Macular OCTA images with corresponding structural sections of the right eye. **(f)** Macular OCTA images with corresponding structural sections of the left eye.

**Figure 4:**
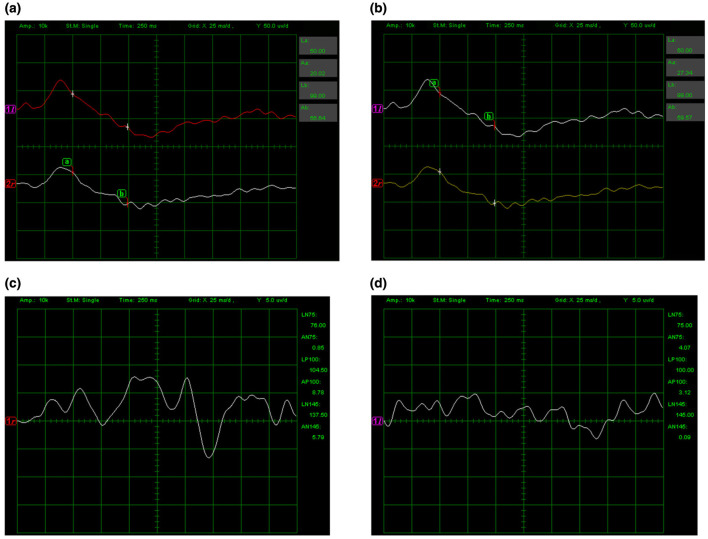
Representative full-field ERG and VEP. Full-field ERG recordings demonstrate no clinically significant abnormalities of a- or b-wave responses in either eye. **(a)** Full-field ERG of the right eye. **(b)** Full-field ERG of the left eye. VEP show preserved P100 latencies in both eyes, with reduced P100 amplitude in the left eye compared with the right eye (right eye: LP100 = 104.50 ms, AP100 = 8.78 μV; left eye: LP100 = 100.00 ms, AP100 = 3.12 μV). **(c)** VEP of the right eye. **(d)** VEP of the left eye. Recordings were obtained with an analysis window of 250 ms and amplifier gain of 10 k. VEP used a white checkerboard stimulus (100% contrast) in single mode at 2.00 Hz (100 sweeps) with band-pass filtering of 0.1–75 Hz (X: 25 ms/div; Y: 5 μV/div). ERG used white flash stimulation in single mode at 0.50 Hz (flash strength 1.125 × 10^−3^ cd·s/m^2^; background off) with band-pass filtering of 0.1–75 Hz (X: 25 ms/div; Y: 50 μV/div).

On admission, uncorrected visual acuity (UCVA) was 20/32 in the right eye and 20/50 in the left eye; best-corrected visual acuity (BCVA) improved to 20/25 in the right eye, with no meaningful improvement in the left eye. Intraocular pressure was within normal limits. Anterior segment examination was largely unremarkable, with mild perilimbal/perikeratic conjunctival injection, clear and intact corneas, deep and quiet anterior chambers, round pupils measuring approximately 3 mm with preserved direct light reflexes, and no obvious lens opacities (Figure 1). Color fundus photography (Carl Zeiss Meditec AG, Jena, Germany), ultra-widefield fundus imaging, and fundus autofluorescence (Optos plc, Dunfermline, UK) revealed no definite abnormalities (Figure 2). Optical coherence tomography (OCT; Heidelberg Engineering, Heidelberg, Germany) demonstrated bilateral, fovea-centered outer retinal/photoreceptor abnormalities in the macular region. The photoreceptor-related bands between the retinal pigment epithelium (RPE) and the outer nuclear layer (ONL) appeared irregular and partially disrupted (e.g., at the level of the ellipsoid/interdigitation zone), accompanied by scattered hyperreflective foci/deposit-like changes within the outer retina. Qualitatively, the abnormalities were present in both eyes and were more conspicuous in the left eye, consistent with the asymmetric visual function. Quantitative measurements on the fovea-centered B-scans (Figure 3) showed a central macular thickness (CMT) of 184 μm in the right eye and 180 μm in the left eye, and an outer retinal thickness (ORT) of 124 μm in the right eye and 136 μm in the left eye. The linear extent of the affected outer retinal segment measured on the scan was approximately 636 μm in the right eye and 720 μm in the left eye. Peripapillary retinal nerve fiber layer (RNFL) thickness maps did not show definite thinning. Optical coherence tomography angiography (OCTA) showed no abnormal macular neovascularization (Figure 3). Full-field flash electroretinography (ERG) was unremarkable, with no prolongation of a- or b-wave implicit times and no clinically significant amplitude abnormalities. Visual evoked potentials (VEP) demonstrated no delay in P100 latency in either eye; the right eye amplitude was within the normal range, whereas the left eye amplitude was decreased, indicating reduced functional responsiveness of the visual pathway or a reduced number of responding neurons (Figure 4). Other ophthalmic examinations, including visual field testing, were normal.

**Figure 5:**
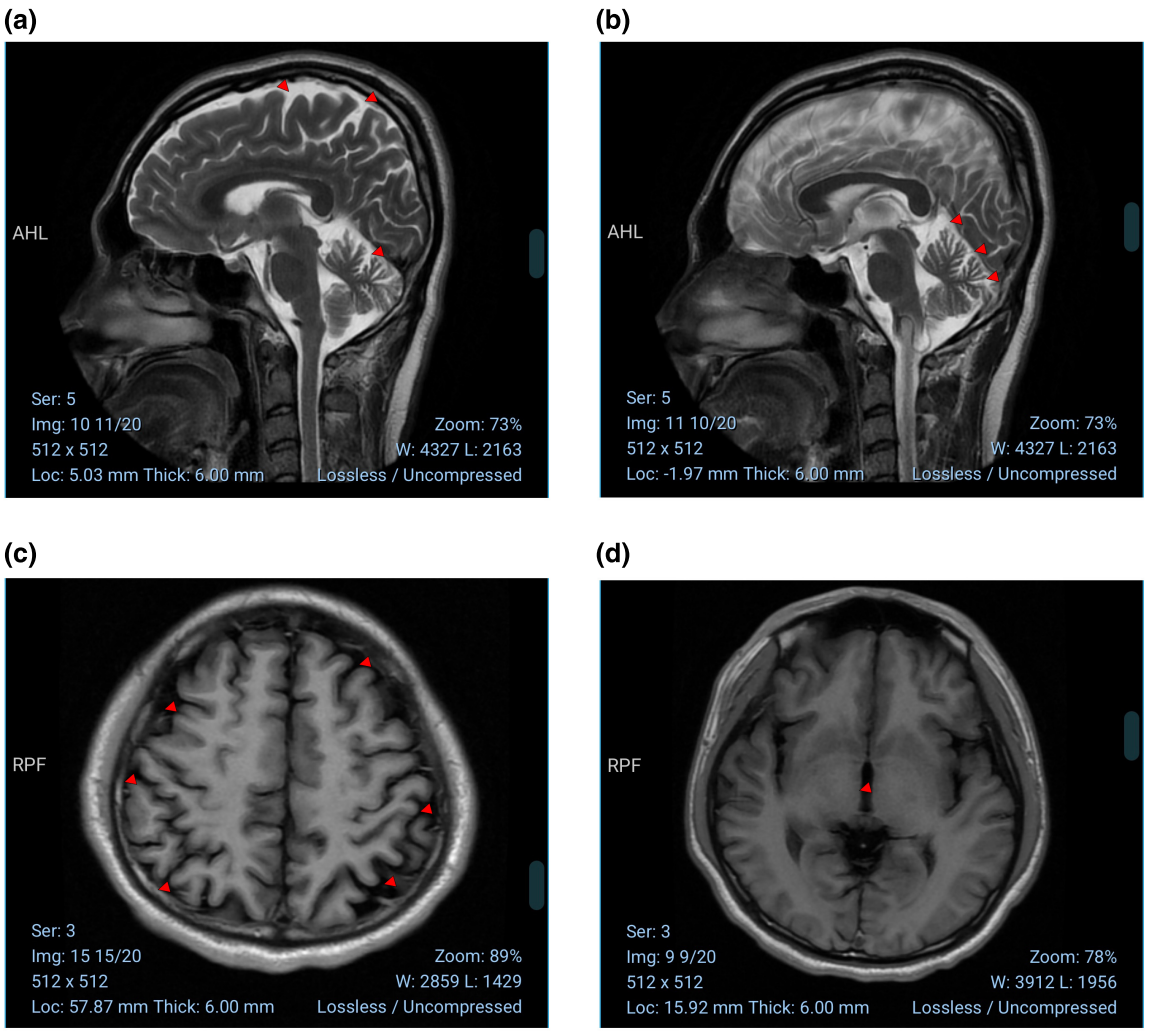
Brain MRI. Brain magnetic resonance imaging shows symmetric cerebral hemispheres with preserved gray–white matter differentiation and no focal abnormal signal. Mild widening of the cerebral sulci and cerebellar fissures is noted, consistent with possible early diffuse cerebral and cerebellar atrophy. **(a, b)** Sagittal MRI sequences. **(c, d)** Axial MRI sequences.

Neurological examination revealed hyperreflexia of the bilateral patellar tendons and dysmetria on finger-to-nose testing, suggesting cerebellar involvement with pyramidal tract signs. The clinical course was slowly progressive, and there was no substantial short-term improvement after discontinuation of tiletamine, raising concern for persistent central nervous system injury potentially related to drug exposure. Brain magnetic resonance imaging (MRI; Figure 5) showed symmetric cerebral hemispheres with preserved gray–white matter differentiation and no focal abnormal signal. The ventricular system and cisterns were essentially normal in morphology and size. Mild widening of the cerebral sulci and cerebellar fissures was noted, consistent with possible early diffuse brain atrophy. Routine hematology and biochemical tests were unremarkable. Transthoracic echocardiography showed trivial regurgitation of the aortic, mitral, and tricuspid valves with preserved left ventricular systolic function. Electrocardiography was unremarkable. Electromyography, nerve conduction studies (motor and sensory conduction velocities), F-wave testing, and bilateral lower-limb somatosensory evoked potentials (SEPs) were within normal limits; no evidence supported a systemic metabolic, infectious, or immune-mediated disorder.

Given the patient-reported history of prolonged non-medical oral exposure to a suspected tiletamine-containing product, progressive visual dysfunction accompanied by cerebellar and pyramidal signs, and multimodal findings—including outer retinal abnormalities on OCT, a preserved full-field ERG with a unilateral reduction in VEP P100 amplitude, and neuroimaging features suggestive of diffuse brain atrophy—the overall presentation was considered most consistent with chronic retinopathy temporally associated with long-term exposure to the suspected agent, with possible central nervous system involvement. A focused differential diagnosis was systematically considered. Although mild conjunctival/perilimbal injection was noted on anterior segment photographs, the patient reported no ocular discomfort (e.g., foreign-body sensation, pain, photophobia, tearing) and slit-lamp examination did not reveal anterior segment inflammation; together with a normal-appearing optic disc and no definite RNFL thinning on OCT, these findings argued against active inflammatory optic neuropathy or ischemic optic neuropathy as primary explanations. Visual field testing was normal, and VEP demonstrated preserved P100 latency bilaterally with a predominantly left-sided reduction in amplitude, a pattern less consistent with acute demyelinating optic neuritis and more compatible with reduced functional responsiveness along the visual pathway. Multimodal retinal imaging (color fundus photography, ultra-widefield imaging, fundus autofluorescence, and OCT/OCTA) revealed no definite fundus lesions or macular neovascularization, whereas OCT showed predominant outer retinal/photoreceptor-level disruption with hyperreflective foci, supporting a retinopathy centered on outer retinal structures rather than a primary optic nerve head process. Given the fovea-centered outer retinal involvement and the absence of inflammatory, vascular, or generalized degenerative signs on the available evaluation, alternative causes of maculopathy/macular atrophy were considered but deemed less likely. With the investigations available, there was no evidence supporting inherited retinal degeneration or a familial neurodegenerative disorder. The patient denied a family history of neurological disorders or inherited retinal degenerative diseases, and fundus examination did not demonstrate typical pigmentary changes. Routine hematology and biochemical tests were unremarkable. Extensive neurophysiological evaluation (electromyography, nerve conduction studies, F-wave testing, and lower-limb SEPs) was normal, arguing against peripheral neuropathy or a systemic metabolic, infectious, or immune-mediated disorder in this clinical context. Brain MRI showed no focal abnormal signal or structural lesion to explain the symptoms, while mild widening of the cerebral sulci and cerebellar fissures suggested possible diffuse atrophy. Taken together, and in the absence of other identified retinotoxic exposures by history, the combined retinal and central nervous system findings were considered most compatible with a chronic toxic/neurotoxic process temporally associated with the patient-reported long-term exposure.

**Figure 6:**
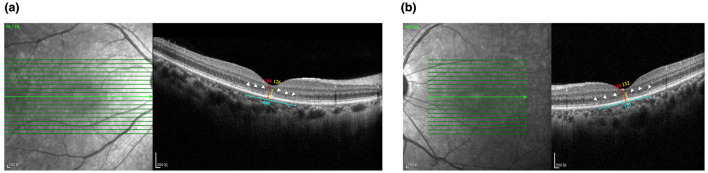
One-month follow-up macular OCT with quantitative measurements. The follow-up OCT scans show persistent, fovea-centered outer retinal/photoreceptor abnormalities (white arrowheads) without obvious qualitative progression compared with baseline. The red vertical line indicates CMT, the yellow vertical line indicates ORT, and the blue horizontal line indicates the linear extent of the affected outer retinal segment. Quantitative values on the shown scans are: CMT 184 μm and ORT 126 μm with an affected-segment length of 640 μm in the right eye; CMT 180 μm and ORT 132 μm with an affected-segment length of 722 μm in the left eye. **(a)** Right eye. **(b)** Left eye.

According to the patient, after discontinuing the suspected agent, he did not experience a marked short-term reversal of either visual or neurological symptoms. Management therefore focused on empirical, symptomatic, and supportive care. A neurotrophic/neuroprotective adjunct, citicoline (tablet), was initiated at 500 mg/day orally. Supportive retinal care targeting oxidative stress included antioxidant supplementation using an AREDS2-based formulation (vitamin C 500 mg, vitamin E 400 IU, lutein 10 mg, zeaxanthin 2 mg, zinc 80 mg, and copper 2 mg; oral, once daily), together with a daily multivitamin tablet (oral, once daily). These agents were continued for 4 weeks until the 1 month follow-up visit. By patient report, adherence was good and no treatment-limiting adverse effects occurred. At the 1 month follow-up visit, best-corrected visual acuity remained unchanged from baseline (20/25 in the right eye and 20/50 in the left eye), and the overall clinical status showed no substantial short-term improvement. Macular OCT remained qualitatively stable without obvious progression of the fovea-centered outer retinal/photoreceptor abnormalities. Quantitative measurements on the follow-up scans showed a central macular thickness of 184 μm in the right eye and 180 μm in the left eye, and an outer retinal thickness of 126 μm in the right eye and 132 μm in the left eye. The measured linear extent of the affected outer retinal segment was approximately 640 μm in the right eye and 722 μm in the left eye (Figure 6). These quantitative values should be interpreted with caution, as they may be influenced by scan positioning, segmentation boundaries, and inherent measurement variability. The patient was advised to return for follow-up every 1–2 months thereafter to monitor visual function and repeat OCT/OCTA, with additional functional testing performed as clinically indicated.

## 3 Discussion

Tiletamine is widely used in veterinary anesthesia, however, systematic evidence regarding the effects of long-term exposure on the human central nervous system and retinal function remains scarce. The existing human literature consists largely of case reports, most of which involve accidental exposure, misuse/abuse, or short-term exposure. For example, a 30-year-old female zoo employee was found unresponsive after recreational injection of Telazol in combination with diazepam and responded only to painful stimuli (17). A 16-year-old adolescent developed dysarthria, gait instability, and seizure-like episodes after oral Telazol, accompanied by ocular signs such as conjunctival injection and nystagmus (18). A 35-year-old male veterinarian experienced central nervous system excitation and involuntary movement disorders after Zoletil injection (19). In addition, acute poisoning and death have been reported in a 45-year-old and a 22-year-old after high-dose injection (5, 20). Collectively, these cases predominantly featured acute manifestations—such as ataxia, respiratory depression, seizures, central nervous system excitation, circulatory dysfunction, and delayed emergence—and were largely related to injection or short-term use, with limited long-term follow-up and minimal mechanistic discussion. In contrast, our patient reported prolonged oral exposure to a suspected tiletamine-containing product (>1 year) and presented primarily with progressive visual dysfunction and neuroimaging features suggestive of diffuse brain atrophy. Given the absence of analytical toxicological confirmation and potential formulation variability, causal inference is limited; nevertheless, the temporal association and multimodal abnormalities raise concern for a possible chronic, cumulative injury pattern involving both the visual system and the central nervous system. Notably, this case is distinguished by a structured neuro-ophthalmic work-up that includes multimodal retinal imaging (ultra-widefield fundus photography, fundus autofluorescence, OCT, and OCTA) and electrophysiology (full-field ERG and VEP), demonstrating a fovea-centered outer retinal/photoreceptor abnormality on OCT with preserved full-field ERG and an asymmetric reduction in VEP amplitude. We further provide a short-term longitudinal assessment with repeat OCT and quantitative macular metrics at 1 month follow-up, adding temporal context to the early trajectory. To our knowledge, such a clinical presentation has rarely been reported, underscoring the rarity of this case and its potential implications for clinical vigilance. From a veterinary pharmacology and toxicology perspective, this case provides a clinically relevant safety signal at the animal–human interface, supporting toxicovigilance, occupational risk communication, and surveillance for diversion or misuse of veterinary anesthetic agents.

Reports of retinal injury associated with NMDA receptor antagonists remain rare. Existing evidence suggests that, under specific conditions, the NMDA receptor antagonist ketamine may exert protective effects on the retina and appears to have a favorable ocular safety profile during short-term clinical use. However, although ketamine literature provides a useful mechanistic framework, tiletamine should not be assumed to be pharmacologically equivalent to ketamine. Tiletamine may have higher apparent potency and a different pharmacokinetic/metabolic profile, particularly when exposure involves a tiletamine–zolazepam preparation. Accordingly, ketamine-based extrapolations should be treated cautiously, and tiletamine-related metabolic and neurotoxic effects as an NMDA receptor antagonist could be distinct or more pronounced (21), potentially contributing to the limited short-term recovery observed in this case. In an animal study, Dourado et al. (8) found that topical low-dose ketamine might confer protection against retinal ischemic injury by attenuating histopathologic damage and reducing apoptosis across multiple retinal layers. In clinical practice, a 5-year retrospective study of 679 pediatric patients undergoing ophthalmic surgery reported that ketamine used as an intravenous anesthetic was safe and effective (22). During the study period, no anesthesia-related serious complications occurred, and no child required unplanned resuscitation or endotracheal intubation. Despite these reassuring safety data in controlled, short-term clinical settings, substantial evidence indicates that long-term, high-dose recreational ketamine abuse can induce significant toxicity across multiple organ systems, indirectly highlighting its potential cumulative systemic risks. A 2021 systematic review including 25 controlled studies found that heavy recreational ketamine use was dose-dependently associated with cognitive impairment, psychiatric disturbances, and gastrointestinal and urinary tract symptoms (23). In an animal model, chronic ketamine administration induced apoptosis of cortical neurons and caused injury to the cerebellum, kidneys, bladder, liver, and heart (24). In addition, long-term abuse has been linked to structural and functional brain alterations, including reduced frontal gray matter volume, compromised white matter integrity, and persistent cognitive deficits and psychiatric symptoms. Notably, these studies of long-term abuse have primarily focused on the nervous, urinary, and hepatobiliary systems and have not directly reported or systematically evaluated retinopathy (25–27). Together, these findings support the concept that NMDA receptor antagonists may show acceptable ocular safety under controlled, short-term anesthesia, yet still carry cumulative neurotoxic risk under prolonged, non-medical exposure—an exposure pattern that is particularly relevant when veterinary agents are misused.

ERG is a key tool for assessing retinal function, and the b-wave largely reflects the activity of bipolar cells and Müller glial cells (28). NMDA receptors are functionally expressed in inner retinal cells, including subsets of bipolar cells, retinal ganglion cells, and Müller glia, where they contribute to excitatory neurotransmission and play an important role in b-wave generation (29–31). Animal studies have shown that direct administration of NMDA and other excitatory agonists such as kainate can selectively injure inner retinal neurons, producing a marked reduction in ERG b-wave amplitude with relative preservation of the a-wave, suggesting that photoreceptors are comparatively resilient whereas inner retinal neurons are more vulnerable (32). In the present case, however, full-field ERG was unremarkable, suggesting that diffuse global retinal dysfunction was not evident at the time of testing. This finding does not exclude clinically meaningful macular disease, because localized fovea-centered outer retinal abnormalities may reduce central vision while remaining below the sensitivity of full-field ERG. As an NMDA receptor antagonist, the effects of tiletamine on retinal function remain unclear. One study comparing the impact of different anesthetic regimens on canine ERG waveforms reported that dogs receiving a tiletamine–zolazepam combination had significantly higher b-wave amplitudes after 5 min of dark adaptation than dogs receiving medetomidine (33). Other veterinary studies (34) have evaluated ocular physiological parameters such as intraocular pressure during tiletamine-zolazepam anesthesia, but chronic retinal toxicity data remains lacking. Rather than directly contradicting our observations, this canine finding may reflect an acute, protocol- and species-dependent modulation of ERG under controlled anesthesia, whereas our case concerns prolonged, non-medical exposure with predominantly macular structural abnormalities on OCT and preserved full-field ERG. Accordingly, chronic exposure—if neurotoxic—may manifest as focal maculopathy and/or post-retinal pathway involvement without producing measurable full-field ERG abnormalities. These mechanistic interpretations remain speculative and require validation in controlled experimental models and longitudinal human observations. Further studies are needed to clarify the long-term retinal and neuro-ophthalmic effects of tiletamine exposure.

Notably, OCT revealed abnormalities in the photoreceptor region between the RPE and the ONL at the fovea, characterized by disorganization of the inner and outer segment architecture accompanied by scattered hyperreflective foci or deposit-like changes. These findings suggest photoreceptor-layer injury or metabolism-related pathology and are consistent with the observed decline in visual function (35). Given that full-field ERG was unremarkable in this patient, the OCT abnormalities may represent a localized macular process that is sufficient to impair central vision yet does not produce a generalized ERG signal change. In this patient, the more pronounced visual acuity loss in the left eye was concordant with the VEP findings, which showed preserved P100 latency with reduced P100 amplitude in the left eye, indicating a reduction in response strength and/or the number of functionally responsive neurons along the afferent visual pathway, rather than a latency-prolonging demyelinating process. Although NMDA signaling primarily involves inner retinal cells, inner retinal dysfunction may secondarily affect outer retinal photoreceptors through several mechanisms, including impaired metabolic and supportive functions of Müller glia, disruption of neuron–glia metabolic coupling, or transsynaptic degeneration. These processes can compromise photoreceptor metabolism and survival, ultimately contributing to outer retinal structural degeneration (36–38). Alternatively, the electrophysiological pattern (preserved ERG with reduced VEP amplitude) may also support a prominent post-retinal component, such as dysfunction along the optic nerve/visual pathway, in addition to macular outer retinal involvement.

Chronic inhibition of NMDA receptor signaling may disrupt synaptic plasticity, metabolism, and neurotrophic support, thereby contributing to structural and functional abnormalities of the central nervous system. Ketamine, an NMDA receptor antagonist with structural and mechanistic similarity to tiletamine (39), has been studied more extensively, and accumulating evidence links long-term ketamine use to brain structural changes. Liao et al. (26) reported that chronic ketamine abuse was associated with a significant reduction in frontal gray matter volume, with duration of use and cumulative dose negatively correlated with frontal gray matter volume. Other studies have described widespread cortical thinning and compromised white matter integrity (40). Some reports have suggested involvement of the hippocampus or parahippocampal regions, although findings in hippocampal structures have been heterogeneous across studies (14, 41, 42). In animal models, prolonged exposure to NMDA receptor antagonists has been shown to induce cognitive decline, impaired motor coordination, and behavioral changes (43), which is broadly compatible with the neurological phenotype observed in our patient. Since initiating oral tiletamine use, the patient developed progressively worsening dysarthria affecting daily communication, as well as gait abnormalities characterized by an ataxic/unsteady gait, difficulty walking in a straight line, hyperreflexia of the bilateral patellar tendons, and dysmetria on finger-to-nose testing. Prior work suggests that chronic suppression of NMDA signaling can reduce neural network plasticity in the cerebellum and prefrontal cortex (44), which may provide mechanistic context for the cerebellar ataxia observed in this case. Although the traditional view emphasizes that excessive NMDA receptor activation may trigger excitotoxic neuronal death via Ca^2+^ overload (45, 46), emerging evidence indicates that both acute overactivation and chronic inhibition can be detrimental. Sustained NMDA receptor hypofunction may lead to maladaptive plasticity, neuronal metabolic disturbances, and neurodegeneration (e.g., hippocampal atrophy). This perspective is consistent with the review by McEwen and Gianaros (47), which highlighted the cumulative adverse effects of chronic stress or prolonged pharmacological exposures on neural plasticity. To date, no reports have described long-term oral use of veterinary tiletamine in humans; nevertheless, the mechanistically related evidence summarized above provides indirect, hypothesis-generating context for investigating the long-term safety of tiletamine exposure. Given the absence of established treatment protocols for suspected tiletamine-related neuro-ophthalmic toxicity, management in this case was empirical and supportive. The chosen agents were extrapolated from their reported neurotrophic and antioxidant roles in other optic neuropathies and retinal disorders (48–50). Chronic NMDA receptor antagonism has been linked to neurotoxicity through impaired mitochondrial function and redox imbalance, which may contribute to oxidative stress in both retinal and central visual pathways. Accordingly, citicoline (500 mg/day, oral) was used as a neurotrophic/neuroprotective adjunct, and an AREDS2-based antioxidant formulation plus a daily multivitamin (oral, once daily) was provided as supportive retinal care. Despite these measures, recovery remained limited at the 1 month follow-up, with unchanged best-corrected visual acuity and no obvious qualitative improvement of fovea-centered outer retinal abnormalities on OCT. This modest response may reflect delayed presentation after prolonged exposure, potential irreversibility of photoreceptor-level injury, and/or persistent post-retinal pathway dysfunction. Future studies should incorporate longer follow-up with standardized functional endpoints and evaluate whether earlier cessation and structured rehabilitation can improve recovery trajectories.

This case highlights a potentially under-recognized public health risk associated with the non-medical acquisition and prolonged use of veterinary anesthetic agents through informal supply chains. In this context, the inability to independently verify the product's identity/composition (no residual sample or packaging and no toxicological confirmation) and the reliance on patient-reported dosing underscore an important safety concern: informal sources may involve uncertain formulations and poorly standardized dosing, which can increase cumulative exposure and delay appropriate medical evaluation. From a toxicovigilance perspective, clinicians should consider early engagement with toxicology services and encourage reporting through appropriate local poison control and/or regulatory channels when suspected veterinary anesthetic exposure is identified, particularly when analytical confirmation is unavailable. In the present case, formal reporting to a poison control center or regulatory authority was not performed; however, the patient was advised to report the suspected exposure and to avoid further access to non-medical sources.

A key limitation is the extreme rarity of this presentation; thus, inferences from a single case require cautious interpretation. Causality is constrained by the absence of analytical toxicological confirmation, such as liquid chromatography-tandem mass spectrometry testing of biological samples or residual products, and by uncertainty regarding the exact formulation and purity of the suspected agent (tiletamine alone vs. tiletamine–zolazepam combinations, possible adulterants), with exposure history based on patient report and therefore subject to recall bias and potential co-exposures. Functional characterization was also incomplete: despite reported dyschromatopsia, quantitative color vision testing was unavailable, and therefore the defect type and indices (e.g., color confusion index) could not be reported; contrast sensitivity was not assessed, and formal patient-reported outcomes were not collected. Follow-up was short (1 month), limiting conclusions on long-term trajectory, and external toxicovigilance context (e.g., poison-control surveillance data) was not available to us. A structured causality tool (e.g., Naranjo scale) was not incorporated, given the lack of verified exposure and rechallenge information. Future work should include longer longitudinal follow-up with standardized functional endpoints (including color vision and contrast sensitivity), multimodal structural metrics (e.g., OCT-based outer retinal measures), pathway-level testing (e.g., VEP), objective exposure confirmation where feasible, and controlled models to clarify downstream consequences of chronic NMDA receptor antagonism.

## 4 Conclusion

This case suggests that long-term oral tiletamine use may be associated with progressive visual dysfunction and neuroimaging evidence of brain atrophy, indicating a potential risk of cumulative neurotoxicity. In line with the “One World, One Health” perspective (51), increased awareness and surveillance regarding misuse of veterinary agents at the animal–human interface are warranted. For individuals with relevant exposure, enhanced risk education, standardized management, and multimodal clinical surveillance with longitudinal follow-up are recommended to reduce the likelihood of irreversible injury.

## Data Availability

The original contributions presented in the study are included in the article/supplementary material, further inquiries can be directed to the corresponding authors.
